# Menthol-enhanced cytotoxicity of cigarette smoke demonstrated in two bioassay models

**DOI:** 10.1186/1617-9625-11-18

**Published:** 2013-08-30

**Authors:** Atsuko Noriyasu, Tadashi Konishi, Shinichi Mochizuki, Kazuo Sakurai, Yutaka Tanaike, Ken Matsuyama, Kazuya Uezu, Tomonori Kawano

**Affiliations:** 1 Faculty of Environmental Engineering, University of Kitakyushu, Kitakyushu, Japan; 2 Oita National College of Technology, Oita, Japan; 3 Center for Fire Science and Technology, Research Institute of Science Technology, Tokyo University of Science, Noda, Chiba, Japan

**Keywords:** Cell toxicity, Cigarette, Menthol, Smoke, Capsules

## Abstract

**Background:**

Cigarette smoke is harmful to human health at both cellular and genetic levels. Recently, a unique bioassay for smoke cytotoxicity using air pollution-sensitive plant cells (tobacco) has been proposed.

**Methods:**

Model plant cells (tobacco Bel-W3 cells) and human cells (alveolar epithelial A549 cells) suspended in fresh culture media were exposed to cigarette smoke sampled after lighting the tip of cigarettes (with vs. without menthol capsules) which were attached to a glass pipe connected to the cell-containing plastic tubes. Control cultures were also assessed.

**Results:**

After exposing tobacco plant cells to cigarette smoke, cell death occurred in a dose-dependent manner. Cell death was significantly enhanced by mentholated smoke, while menthol alone was shown to be inert suggesting that menthol synergistically contributes to the enhancement of cell death, initiated by smoke-associated compounds. The enhanced toxicity of mentholated smoke was confirmed in human alveolar epithelial A549 cells.

**Conclusions:**

Cigarette smoke cytotoxicity leading to cell death assessed in plant and human model cells was enhanced by menthol. Further research into these findings is encouraged.

## Introduction

Study on the health effects of menthol cigarettes as compared to non-menthol cigarettes is an area of significant scientific interest as the sales of mentholated cigarettes in the US have increased considerably over the past 50 years, while menthol cigarettes are disproportionately used by youth [1-4]. Moreover, the existing literature suggests that mentholated cigarettes may be perceived as safer than non-mentholated cigarettes [5].

Cigarette smoke is known to be toxic and harmful to human health [6], both at cellular [7] and genetic levels [8]. Since cigarette smoke is derived from combustion of tobacco leaves, the chemical components in the smoke must be the mixture of (i) chemicals originally present in the tobacco leaves and (ii) the chemicals formed through combustion of the industrialized cigarette [9]. We have previously proposed a unique bioassay for cigarette smoke cytotoxicity using the cells of tobacco (*Nicotiana tabacum* L.) [10]. By employing the air pollution-sensitive Bel-W3 tobacco cells as model materials for cigarette smoke toxicity assay, the impact of combustion by-products such as nitrogen oxides can be highlighted while the toxic impact of the chemicals native to tobacco plants such as nicotine [11] and phenolics [12] could be minimized or excluded in cell death mechanisms [10].

Our previous research suggested that cigarette smoke effectively induces apoptosis-like cell death in tobacco plant cells through the involvement of combustion-derived nitric oxide (NO) as one of cell death mediators [10]. Use of plant materials as the models for human health is not surprisingly new, as many discoveries with direct relevance to human health and disease have been elaborated using plant models, and several processes important to human biology are more easily studied in these versatile model plants possibly with higher sensitivity [13]. Following the assays with plant cells, it is also encouraged to testify or confirm the toxicity of the cigarette smoke and related compounds using the human or animal lung-derived cells in order to discuss the impact on human health. Use of *in vitro*-cultured human alveolar epithelial cells (A549) is an established cytotoxicity assay model for assessing the impact of direct whole smoke exposure [14].

In this short report, we compared the lethal impacts of non mentholated vs. mentholated (capsule) cigarette smoke in both tobacco Bel-W3 cells and human A549 cells.

## Materials and methods

As previously described [10], the suspension cultures of tobacco Bel-W3 cells (5 day-old culture) were propagated at 23°C in Murashige-Skoog liquid medium with 2,4-D. Human A549 cells were propagated under 5% CO_2_ at 37°C using the growth medium with F12/K (Gibco/Invitrogen), 10% fetal bovine serum, 100 units/ml penicillin, and 100 μg/ml streptomycin as reported [14].

Commercially available packs of cigarettes equipped with the menthol capsule-embedded filters (Japan Tobacco Inc., Tokyo) and non-menthol cigarettes were purchased from a local vender. Within the filter of each cigarette, a ball-shaped capsule of menthol “ice ball” is embedded. We conducted, 4 types of experiments comparing (i) the air, (ii) smoke from non-mentholated products, (iii) smoke from menthol capsule-equipped products without crushing the capsule (thus non-menthoated), and (iv) smoke with intense menthol flavor after crushing the menthol capsules.

The aforementioned model cells suspended in the fresh culture media were exposed to cigarette smoke using a set of apparatuses described previously [10]. Cigarette smoke was prepared by igniting the tip of the cigarettes (with filters) connected to the glass pipe connected to the sealed plastic test tube (50 ml) containing 10 ml of cell suspension. Cigarette smoke was loaded into the plastic tube by aspirating the air at 1.5 L/min using an air pump (HiBlow 3EBS, Kenis Kagaku Kyoeisha Ltd., Tokyo, Japan). When combustion of a cigarette was completed, additional cigarettes were used for serial exposure. Thus, the extent (dose) of smoke exposure was adjusted by the number of cigarettes used for serial exposure. As the time required for combustion of single cigarette was no-longer than 90 second in our setup, cigarettes were replaced every 90 seconds. As a control, model plant and human cells were exposed to air passed through cigarettes without lighting.

The impact of smoke exposure was determined by monitoring the level of cell death induction according to previous reports [10]. The lethal impact of cigarette smoke exposure was quantified by counting dead cells. The vital stains used for plant and human cell assays were Evans blue and trypan blue, respectively. Then stained cells were observed under microscopes (SMZ800, Nikon, Tokyo, Japan). For means of analysis, 3–4 different digital images of cells under the microscope (each covering 50–100 cells to be counted) were acquired and stained cells were counted. The changes in the chemical composition of the sampled post-combustion gas were monitored with Fourier transform infrared (FTIR) spectroscopy.

## Results

FTIR smoke analysis of both cigarettes (non mentholated and mentholated without capsule rupture) revealed the approximate levels (ppm) of major hydrocarbons such as CH_4_ (480), C_3_H_4_O (375), C_3_H_8_ (120), C_2_H_4_ (60), C_2_H_6_ (300), C_6_H_14_ (55), C_6_H_6_O (1.5), and CH_2_O (0.5), and inorganic gases such as CO (5000), HBr (200), HCN (75), SO_2_ (30), HCl (25), NH_3_ (10), NO (10), NO_2_ (8), N_2_O (7), and HF (0.1). There was no significant difference between the menthol-added and control smoke samples.

**Figure 1:**
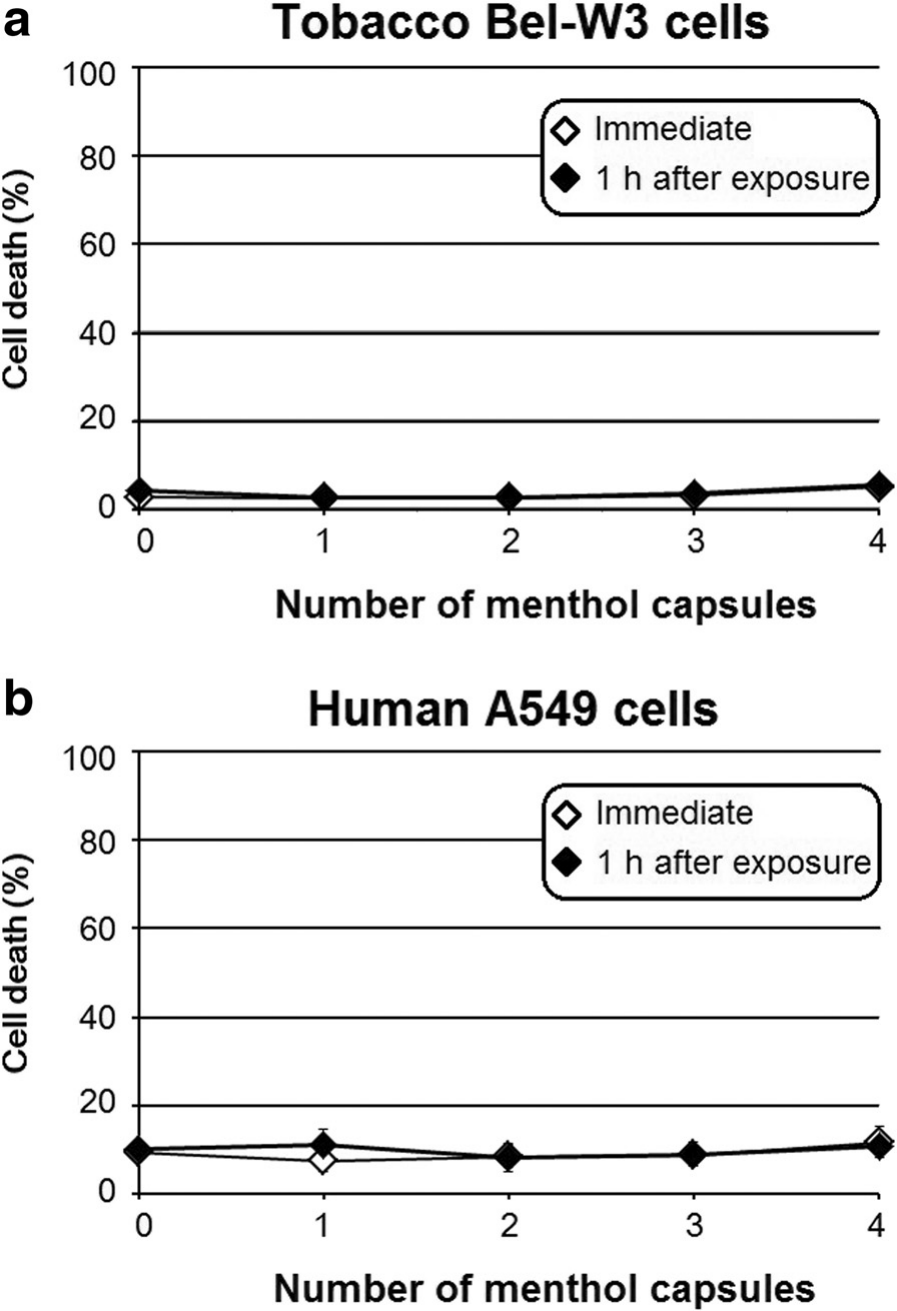
Cytotoxicity of mentholated air without combustion observed on tobacco Bel-W3 cells (a) and human A549 cells (b).

Control exposure to air -passed through a non ignited cigarette- induced no increase in cell death in both model cell types (data not shown). Similarly, no cytotoxicity of mentholated air without combustion was observed as the cells of tobacco Bel-W3 cells (Figure 1a) and human A549 cells (Figure 1b) were exposed to mentholated air by crushing the menthol capsules embedded within the filter of the cigarettes.

**Figure 2:**
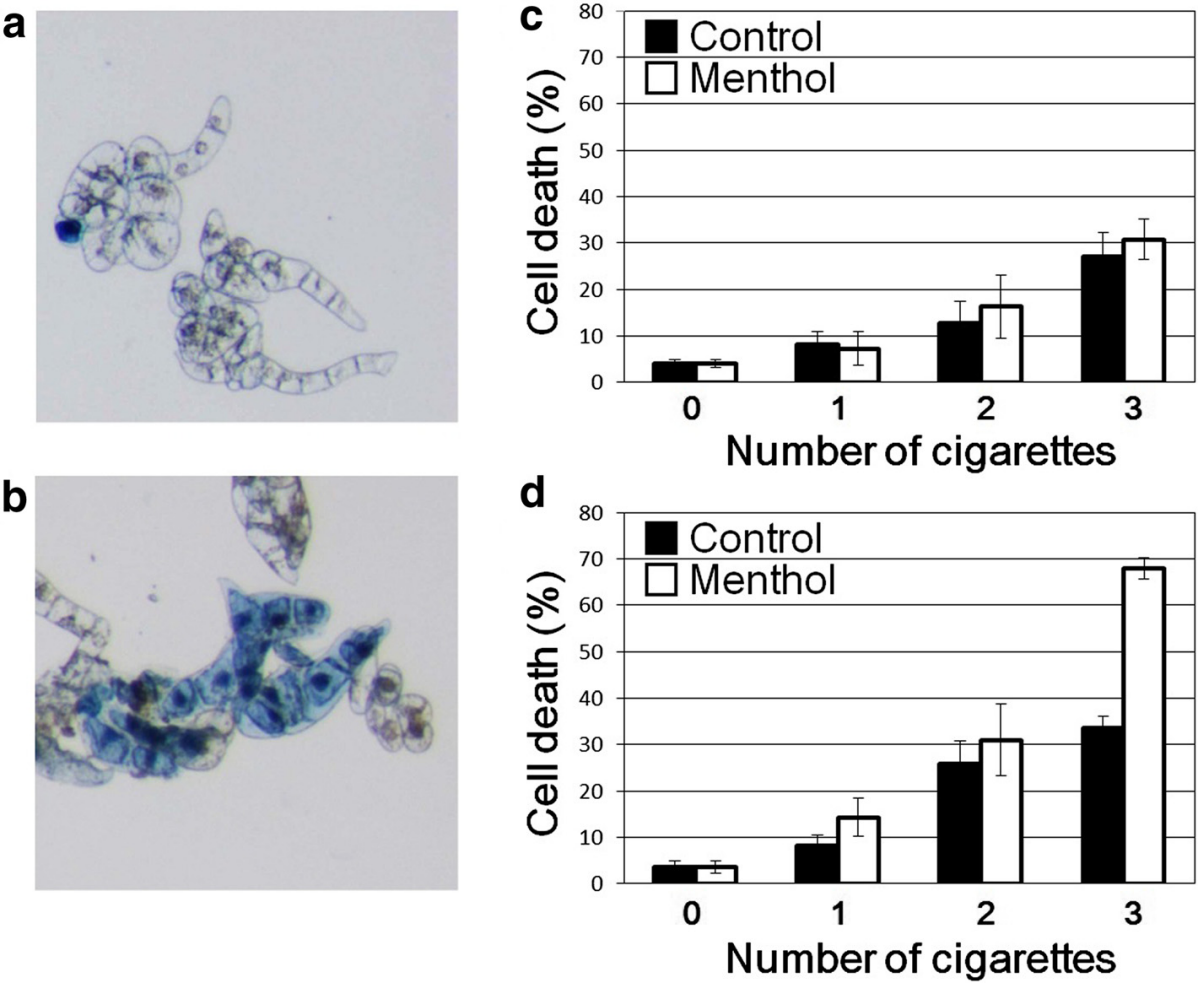
**Effect of cigarette smoke on cell death induction in tobacco plant Bel-W3 cells.** Typical images of vital staining before **(a)** and after **(b)** exposure to smoke. Cell death was quantified immediately after exposure to smoke **(c)** and after further incubation for 1 h **(d)**. Each data point represents the mean of 4 replicates (each, 100 cells; bars, S.D).

**Figure 3:**
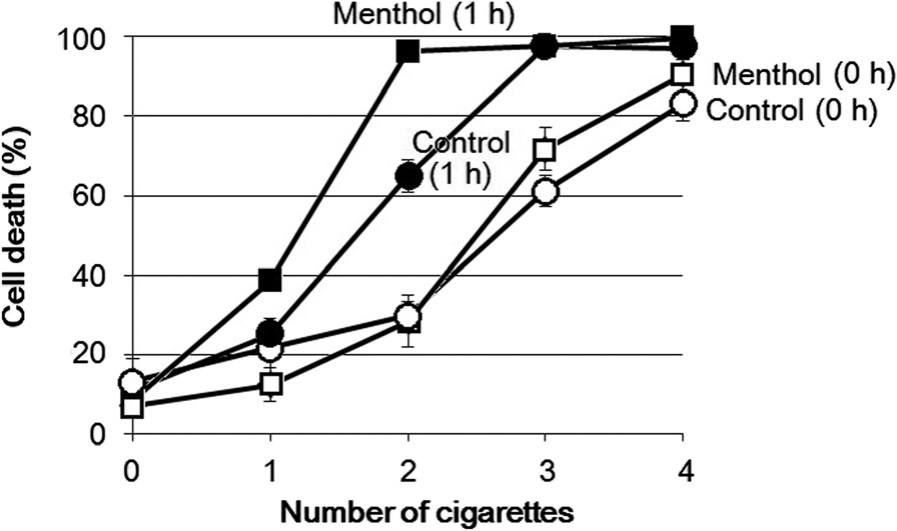
**Effect of cigarette smoke on cell death induction in A549 cells.** Cell death was quantified immediately after the exposure to smoke and after further incubation for 1 h. Each data point represents the mean of 4 replicates (each, 100 cells; bars, S.D).

By exposing the tobacco cell suspensions to the cigarette smoke, we observed the gradual development of cell death depending on the dose of smoke exposure (number of cigarettes used). Interestingly, the higher level of cell death was observed after exposure to the mentholated smoke (Figure 2), while menthol alone (without smoke) was inert. Treatment (iv) showed enhanced toxicity compared to treatments (ii and iii). For clear-cut comparison, only (iii) and (iv) are compared in the Figures 2 and 3. This may imply that menthol synergistically contributes to the enhancement of cell death development, initiated by smoke-associated compounds.

Lastly, we confirmed the enhanced toxicity of mentholated cigarette smoke also in human A549 cells. Cell death in A549 cells were significantly enhanced by mentholated smoke compared to non-mentholated cigarette smoke (Figure 3), although the human cell response to the mentholated smoke was less obvious when compared to the plant cells’ response.

## Discussion

To date, a number of researches have focused on nicotine dependence as tobacco use is basically driven by dependence on nicotine. Furthermore, a few researches have recently started to highlight the role of menthol flavor in nicotine dependence [15]. In addition to the role of menthol enhancing the addiction to cigarettes, the present study revealed that menthol might enhance the toxicity of cigarette smoke.

We demonstrated that mentholated cigarette smoke showed higher toxicity to model plant and human cells while mentholated air without combustion was shown to be inert. Our previous report [10] suggested that a plant cell model may serve as a smoke toxicity model that may be compared with a human cell model. As expected, FTIR smoke analysis system detected the presence of NO in the cigarette smoke which may suggest that combustion-derived NO may partially contribute to the toxicity of cigarette smoke. However, NO levels did not significantly differ between smoke from mentholated and non-mentholated cigarettes. Therefore, it is likely that the addition of menthol may alter the sensitivity of the living plant and human cells to the smoke components, but not the chemical compositions of the combustion gasses. As menthol has been shown to have analgesic, cooling, and muscle relaxing activities by its effect on transient receptor potential cation channel subfamily M member 8 (TRPM8), Kappa receptors stimulation, and inhibition of voltage-gated sodium channels [16,17]. It is tempting to speculate that the menthol-sensitive receptors in both plant cells [18] and animal cells [19] could be putative factors involved in menthol-enhanced toxicity of cigarette smoke. This point must be clarified in the future studies.

### Strengths and limitations

Our assay system has several strengths which are the use of model cells reflecting the responses of both plant and animal cells. Use of live tobacco plant cells often aids the differentiation between the toxicity of the plant-derived chemicals, such as nicotine, and the toxicity of the chemicals in the actual cigarette and derived from the combustion process, while the use of human alveolar epithelial cells (A549) helps provide a simple and rapid assessment of the effect of the toxic combination of smoke-derived chemicals and flavoring reagents. While a possible enhancement of smoke toxicity by menthol was suggested by our simple model assays; further biochemical, physiological or population-based studies concerning the health impact of mentholated cigarette smoke are encouraged.

## Conclusions

By exposing the suspension of cultured tobacco plant cells to cigarette smoke, acute cell death was induced in a dose-dependent manner. Interestingly, this smoke-induced cell death was further enhanced by exposure to menthol. Moreover, the menthol-dependent toxicity of cigarette smoke was further confirmed in human alveolar epithelial A549 cells. Since menthol alone was shown to be inert to both plant and human model cells, we concluded that mentholated smoke synergistically damages the cells through a mechanism potentially common to both plants and animals.

## Competing interests

The authors declare that they have no competing interest.

## Authors’ contributions

AN, SM, KS and Konishi contributed to bioassays. YT, KM, KU technically contributed to smoke analyses. Kawano designed, directed and joined the experiments. All authors read and approved the final manuscript.
